# Effect of adding flaxseed powder to Awassi sheep diets on reproductive performance and progesterone and estrogen levels in the blood

**DOI:** 10.1016/j.vas.2026.100936

**Published:** 2026-11-06

**Authors:** Mohamed Alrez, Rayyan Terro

**Affiliations:** a Department of Animal Production-Faculty of Veterinary Medicine - University of Hama, Hama city, Syria; b Department of Public Health and Preventive Medicine-Faculty of Veterinary Medicine - University of Hama, Hama city, Syria

**Keywords:** Awassi sheep, Estrogen, Flax seeds, Progesterone, Reproductive performance

## Abstract

**Background:**

Good nutrition and the use of effective feed additives are important factors in improving reproductive efficiency. Therefore, the study aimed to determine the effect of using different percentages of flaxseed powder on the reproductive efficiency of Awassi sheep and the concentrations of progesterone and oestrogen hormones in the blood. In the experiment, forty-eight ewes of similar age and body weight were used. The ewes were distributed into four groups according to the content of flaxseed powder in the diet: Group T1 (control) was fed a diet that did not contain flaxseed powder, Group T2 was fed a diet containing 3% flaxseed powder, while Group T3 was fed a diet containing 5% flaxseed powder, and Group T4 was fed a diet containing 7% flaxseed powder. The experiment lasted for 6 months during the breeding season.

**Results:**

The results indicated a significant increase (*P* ≤ 0.05) in the estrus rate (75%, 75%, and 83.33%, respectively) and the duration of estrus (33.35, 35.32, and 36.22 h, respectively) in the treatment groups compared to the control group T1 (*58.33*%,29.44 h). The treatment groups also achieved a significant increase (*P* ≤ 0.05) in the pregnancy rate (77.78%, 88.89%, and 90%), Due to the occurrence of twin births, lambing rate reached (128.57%, 137.5%, and 144.44%), and the twinning rate (28.57%, 37.5%, and 44.44%, respectively) compared to the control group T1(*71.43*%,*120*%,20%). Furthermore, the results showed a significant improvement (*P* ≤ 0.05) in the serum progesterone and oestrogen levels of the ewes in the treatment groups during the experimental period. However, there was no significant effect (*P* > 0.05) of the treatments on lamb weights after birth.

**Conclusions:**

These preliminary findings suggest that dietary supplementation with flaxseed powder may potentially enhance reproductive performance and elevate serum concentrations of progesterone and oestrogen in Awassi sheep. However, further confirmation is required through large-scale future studies

## Highlights

• Adding flaxseed powder to Awassi sheep feed improved the onset and duration of estrus.
• Adding flaxseed powder contributed to improved pregnancy rates, lambing rates, and twin births.
• Adding flaxseed played a role in improving reproductive hormone balance and promoting pregnancy.
• Adding flaxseed at a rate of 7% to the feed achieved the best results in improving the reproductive performance of ewes.

## 1 Background

Sheep are a major source of high-quality animal protein Almohsen et al. (2026), and nutrition plays a crucial role in increasing sheep productivity and improving their reproductive performance (Alrez, 2025). The use of flaxseed powder in rations is gaining increasing attention due to its richness in beneficial nutrients that contribute to enhancing the health and physiological functions of ewes (Habeeb, 2025). flaxseed powder is a food source containing high levels of essential fatty acids, particularly omega-3 fatty acids (alpha-linolenic acid) (Ulfina et al., 2015), in addition to high-quality proteins, dietary fiber, and antioxidant compounds such as phenols and lignans, which are phytoestrogens with antioxidant properties that may affect hormonal balance (Cui et al.*,* 2022). These components contribute to promoting the overall health of sheep and improving their productive performance (Vaisey-Genser & Morris, 2003). Omega-3 fatty acids support nervous system development and tissue growth in animals, and contribute to improved meat quality. The protein content in flaxseed powder increases the nutritional value of feed, positively impacting growth rates and weight gain Cui et al. (2022). Fiber and plant compounds also play a role in improving digestive function and rumen microflora balance, and enhance nutrient absorption (Shim et al.*,* 2015).

Several studies have indicated that adding flaxseed powder to feed rations plays a significant role in improving reproductive efficiency. Moats (2015) found that adding flaxseed powder to feed improved animal reproductive efficiency and increased embryo survival in early stages.

Furthermore, flaxseed powder supplementation has been shown to promote early return to estrus, uterine recovery, and early fertilization, leading researchers to conclude that flaxseed powder can be safely added to diets to regulate reproductive processes (Ulfina et al.*,* 2015). Jahani-Moghadam et al. (2015) also noted that flaxseed powder consumption resulted in a reduction in postpartum bleeding, a decrease in the incidence of polycystic ovaries, and improved energy balance, potentially influencing the reactivation of the pituitary and hypothalamic glands, and consequently, ovarian function. Furthermore, increased dietary fat intake has direct effects on ovarian structure (Dirandeh et al., 2013a, 2013b, 2015). Feeding cows (Esposito et al.*,* 2014) and sheep (Scaramuzzi et al.*,* 2006) fatty acids improves their reproductive performance (Fouladi-Nashta et al., 2009). It alters the fatty acid composition of follicular fluid and granulosa cells, enhancing the interaction between ligands and their membrane receptors, as well as promoting gene expression. This leads to improved follicular formation, egg cytoplasmic maturation, steroidogenesis, ovulation rate, and increased embryo survival and gestational age (Ababakri et al., 2021).

## 2 Importance and objectives

Given the high nutritional value of animal protein and the continuous human need for it, and the role that nutrition through the use of natural food additives may play in reducing reliance on hormonal treatments in improving reproductive efficiency in sheep, this research aimed to study the effect of adding flaxseed powder to the diets of Awassi sheep on some reproductive indicators and blood levels of progesterone and oestrogen hormones, in addition to the weights of lambs after birth.

## 3 Materials and methods

### 3.1 Study setting and experimental design

The experiment was conducted from the beginning of August to the end of January 2026, lasting six months, on a private sheep farm in the town of Besirin in the southern Hama countryside, at an altitude of approximately 417 m above sea level. The climate is generally a semi-arid Mediterranean, with hot, dry summers and relatively mild to cold winters with seasonal rainfall.

### 3.2 Experimental animals and rearing and housing conditions

Forty-eight Awassi ewes were used in the experiment, with an average weight of 45 ± 2 kg and similar ages (3–4 years). Each ewe had given birth at least once and had not received hormonal treatment for more than five months.

The ewes were divided equally, according to age and weight, into four groups of twelve ewes each. This division was designed to increase the statistical accuracy of the results, reduce random variation within a single treatment, and compensate for any loss or unforeseen exclusion of ewes. Each group was housed in one of four separate pens, with each pen divided by wire mesh into individual stalls for each ewe to isolate them from one another. The pens were equipped with individual waterers and feeders according to health standards, and the ewes were tagged to facilitate monitoring and recording of results. They were also vaccinated according to the vaccination program approved by the Syrian Ministry of Agriculture. Before the start of the experiment, the ewes underwent a ten-day acclimatization period to the new diet, during which the amount of flaxseed powder in their ration was gradually increased.

### 3.3 Feeding rations

The rations were individually provided to the ewes in each group to ensure each ewe received its allocated amount, thus increasing the accuracy of the group data. The rations were a concentrated feed that met their nutritional requirements according to the recommendations of the National Research Council (1985), and the chemical analyses of the feed were performed according to AOAC (2002), and were designed to be as similar in nutrient levels as possible to demonstrate the effect of flaxseed powder, as shown in Table 1. The first group (control) was fed a contained 0% flaxseed powder. The sheep in the treatment groups (groups two, three, and four) were fed concentrated rations containing 3%, 5%, and 7% flaxseed powder, respectively. Wheat straw was freely provided to the animals at a rate of 400 g per ewe daily at 7:00 AM and 4:00 PM, and clean water was provided freely throughout the study period.

**Table 1:** Ingredients and chemical composition of experimental diets.

**Items**	Experimental concentrate diets %				
	T1 Control	T2	T3	T4	
**Ingredients**					
Barley	64	64	64	64	
Bran	15	12	10	8	
Decorticated cottonseed	18	18	18	18	
flaxseed powder	0	3	5	7	
Salt	1	1	1	1	
Calcium carbonate	1	1	1	1	
Vitamins and minerals^1^	1	1	1	1	
Percentage	100	100	100	100	
**Chemical composition**					**wheat straw**
Dry Matter	89.13	90.86	91.23	91.46	91
Organic Matter	95.85	96.1	96.85	97.25	93.3
Crude Protein	15.23	15.65	15.73	15.95	4.2
Ether Extract	3.09	3.55	3.79	4.15	1.4
Crude Fiber	7.57	7.35	7.19	6.87	41.5
Ash	4.15	3.9	3.15	2.75	6.7
Nitrogen Free Extract	66.23	67.1	67.93	68.2	46.2
Metabolizable Energy kcal/kg	2576	2580	2596	2612	997

T1: Control group: Feed that does not contain 0% flaxseed powder. T2: Feed containing 3% flaxseed powder.

T3: Feed containing 5% flaxseed powder. T4: Feed containing 7% flaxseed powder.

1 Each kg contained: vitamin A (2000,000 IU), vitamin D3 (500,000 IU), vitamin E (2500 mg), iron sulfate (5000 mg), manganese sulfate (1000 mg), copper sulfate (3000 mg), potassium iodide (100 mg), zinc sulfate (5000 mg), cobalt sulfate (50 mg), slinat sodium (100 mg), sodium chloride (25,000 mg).

### 3.4 Estrus detection and insemination

Five rams were selected after their health, vitality, and fertility were assessed. The wool of the rams and ewes was sheared around the genital area (and hooves were trimmed) to facilitate mounting. The ewes in the isolated groups were inseminated four weeks prior to the start of the experiment. At the end of the feeding period, on day 60, a scout ram was equipped with a special estrus detection apron with colored dye and introduced to the groups twice daily, between 6:00 and 7:00 AM and between 5:00 and 6:00 PM. The estrous ewes were isolated and inseminated with four rams (one ram / group). The onset and end times of estrus for each ewe were recorded, the duration of the estrous cycle was calculated, and the percentage of ewes exhibiting signs of estrus was determined as follows:

Rate of estrus = Number of ewes in estrus / Total number of ewes * 100
Estrus induction interval = Total length of estrus / Number of ewes in estrus (Ngcobo et al., 2023)

### 3.5 Pregnancy, births, and lamb weights

Thirty-five days after insemination, pregnancy was diagnosed using a 7.5 MHz ultrasound (Mindray, USA), and the percentage of pregnant ewes was calculated. The ewes were followed and monitored until lambing, and the number of lambs, twin births, single births, and total births were recorded. Lamb weights were recorded on the day of birth, after 7 days, and after 15 days using an electronic scale. Reproductive indicators for each group were calculated as follows:

Pregnancy rate = Number of pregnant ewes / Total number of ewes mated * 100.
Twin birth rate = Number of ewes giving birth to twins / Total number of ewes lambing * 100.
Single birth rate = Number of ewes giving birth to single / Total number of ewes lambing * 100.
Lambing rate = Number of lambs born / Total number of ewes lambing* 100 (Ngcobo et al., 2023).

Blood samples were collected on days −10, 0, 15, 30, 45, and 60 of the experiment between 7:00 and 8:00 am via jugular vein using sterile medical syringes and stored in vacuum-sealed tubes containing coagulation-activating factors to obtain serum. On day 18 post-estrus, blood samples were collected from the animals (day zero of the estrous cycle) to determine progesterone concentration. These samples were centrifuged at 3000 rpm for 10 min using a centrifuge (KL04A-II, Hunan Kaida Scientific Instrument Co., Ltd., Hunan, China) to obtain serum. The serum samples were then stored at −20 °C until analysis (Alrez et al., 2024).

### 3.6 Hormonal analysis

#### 3.6.1 Serum progesterone concentration

Serum progesterone concentration (ng/mL) was measured on six different days (−10, 0, 15, 30, 45, and 60) during the experiment using an ELISA kit from Cayman Chemicals, USA (582,601). The test range was 7.8–1000 pg/mL, and its sensitivity was approximately 10 pg/mL (80% B/B0).

#### 3.6.2 Serum estradiol concentration

I125 diagnostic kits from Immunotech (France and BARC, Mumbai, India) were used to estimate oestrogen concentration in serum samples. The analytical sensitivity of the kit for estradiol estimation was <6 pg/mL. The intra-test and inter-test coefficients of variation for estradiol were 12.1% and 11.2%, respectively.

### 3.7 Statistical analysis

The experimental data were statistically analyzed using SPSS Statistics (IBM®) version 26. For categorical and binary variables, the Generalized Linear Model (GLM) was used. For continuous variables measured repeatedly over time (progesterone and oestrogen concentrations), the Linear Mixed Model (LMM) was used. This model included fixed effects: treatment, time, and the treatment-time interaction. A single ewe was considered a random subject. Significant main effects or interactions between factors were investigated, and multiple comparisons were performed to separate means using Tukey's HSD test. Statistical significance was established at a probability level of 0.05.

## 4 Results

The results shown in Table 2 showed a significant increase (*P* ≤ 0.05) in the percentage of estrus in ewes fed on rations containing flaxseeds powder in groups T2, T3, and T4, reaching 75, 75, and 83.33% respectively, compared to the sheep in the control group T1, which reached 58.33%. The results also showed a significant increase in the length of the estrus period in the groups fed on flaxseeds powder, reaching 35.33, 35.32, and 36.22 h respectively, compared to the sheep in the control group T1, which reached 29.44 h.

**Table 2:** Effect of adding different levels of flaxseed powder to feed rations on estrus in Awassi sheep.

**Studied Characteristics**	**Experimental groups (Mean ± SD)**				***p*-value**
	**T1** Control Flaxseed (0%)	**T2** Flaxseed (3%)	**T3** Flaxseed (5%)	**T4** Flaxseed (7%)	
Rate of estrus *(%)*	(7/12) 58.33^c^	(9/12) 75^b^	(9/12) 75^b^	(10/12) 83.33^a^	0.043
Estrus duration (hours)	29.44±2.19^b^	33.35±2.24^a^	35.32±2.77^a^	36.22±3.54^a^	0.049

Different letters a, b, c within the same line indicates significant differences between groups at a level of (*P* ≤ 0.05).

The results in Table 3 show a significant increase (*P* ≤ 0.05) in the percentage of pregnant ewes in the groups fed flaxseed powder diets (T2, T3, and T4). Group T4 achieved the best results, with pregnancy rates of *77.78*%, *88.89*%, and *90*%, respectively, compared to *71.43*% in the control group (T1).

**Table 3:** Effect of adding different levels of flaxseed powder to feed rations on pregnancy rates, Lambing and twin births in Awassi sheep.

Studied Characteristics (%)	Experimental groups (Mean ± SD)				*p*-value
	T1 Control Flaxseed (0%)	T2 Flaxseed (3%)	T3 Flaxseed (5%)	T4 Flaxseed (7%)	
Pregnancy rate	(5/7) 71.43^b^	(7/9) 77.78^ab^	(8/9) 88.89^a^	(9/10) 90^a^	0.032
Lambing rate	(6/5) 120^b^	(9/7) 128.57^ab^	(11/8) 137.5^a^	(13/9) 144.44^a^	0.027
Twin birth	(1/5) 20^c^	(2/7) 28.57^b^	(3/8) 37.5^a^	(4/9) 44.44^a^	0.015
Single birth	(4/5) 80^bc^	(5/7) 71.43^bc^	(5/8) 62.5^ab^	(5/9) 55.56^a^	0.019

Different letters a, b, c within the same line indicates significant differences between groups at a level of (*P* ≤ 0.05).

The results also show a significant increase (*P* ≤ 0.05) in the lambing rate among ewes in the groups fed flaxseed powder diets (T2, T3, and T4), reaching *128.57*%, *137.5*%, and*144.44*%, respectively, compared to *120*% in the control group (T1). Furthermore, the results indicate a significant increase (*P* ≤ 0.05) in the percentage of twin births in the groups fed flaxseed powder diets, reaching 28.57%, 37.5%, and 44.44%, compared to 20% in the control group (T1). A significant decrease (*P* ≤ 0.05) was observed in the percentage of single births in the treated groups, reaching *71.43*%, 62*.*5%, and 55.56%, respectively, compared to *80*% in the control group.

Table 4 shows the mean serum progesterone concentration (P4) in the control and treatment groups, measured on different days. In groups T2, T3, and T4, serum progesterone concentration was significantly higher (*P* < 0.05) on day 60 of treatment compared to days 10, 0, 15, 30, and 45. Serum progesterone concentration increased significantly (*P* < 0.05) in all treatment groups starting on day 15.

**Table 4:** Effect of adding different levels of flaxseed powder to feed rations on serum progesterone concentration in ewes. (ng/ml).

Groups	Days						*p-value*		
	10-	0	15	30	45	60	Groups	Days	Group x Days
**T1** Control Flaxseed (0%)	±0.02 0.55 Aa	±0.03 0.33 Aa	±0.02 0.45 Bb	±0.34 1.24 Bb	±0.03 1.36 Bb	±0.063.51 Bb	0.002	0.042	0.053
**T2** Flaxseed (3%)	0.65 ± 0.01 Aa	0.87 ± 0.31 Aa	1.19 ± 0.04 Aa	1.92 ± 0.30 Aa	3.02 ± 0.70 Aa	7.13 ± 0.26 Ab	0.059	0.039	0.027
**T3** Flaxseed (5%)	0.56 ± 0.09 Aa	0.73 ± 0.15 Aa	1.28 ± 0.07 Aa	1.53 ± 0.14 Aa	4.03 ± 0.13 Aa	8.43 ± 0.15 Ab	0.041	0.045	0.002
**T4** Flaxseed (7%)	0.36 ± 0.02 Aa	0.43 ± 0.18 Aa	1.08 ± 0.01 Aa	2.16 ± 0.81 Aa	3.61 ± 0.35 Aa	7.38 ± 0.55 Ab	0.021	0.041	0.003

different letters a, b horizontally and A, B vertically indicate significant differences (*P* ≤ 0.05) between the groups.

Table 5 shows the mean serum oestrogen (E2) concentration in the sheep groups, measured on different days. No, statistically significant differences were observed in the mean serum oestrogen concentration in the first group between the sampling days (*P* > 0.05). On treatment days 15, 30, and 45, serum oestrogen concentration was significantly higher (*P* < 0.05) in all treatment groups compared to the control group.

**Table 5:** Effect of adding different levels of flaxseed powder to feed rations on serum oestrogen concentration in ewes. (pg/ml).

Groups	Days						*p*-value		
	10-	0	15	30	45	60	Groups	Days	Group x Days
**T1** Control Flaxseed (0%)	±0.04 3.41 Aa	±0.03 3.36 Aa	±0.34 3.69 Ba	±0.12 3.95 Ba	±0.77 4.08 Ba	±0.52 2.55 Aa	0.025	0.057	0.032
**T2** Flaxseed (3%)	3.63 ± 0.16 Aa	3.72 ± 0.70 Aa	6.42 ± 0.80 Ab	7.19 ± 0.34 Ab	6.17 ± 1.31 Ab	3.05 ± 0.52 Aa	0.032	0.032	0.033
**T3** Flaxseed (5%)	3.43 ± 0.15 Aa	4.03 ± 0.13 Aa	7.13 ± 1.14 Ab	8.78 ± 1.14 Ab	6.73 ± 1.15 Ab	3.14 ± 0.59 Aa	0.021	0.023	0.019
**T4** Flaxseed (7%)	3.38± 0.25 Aa	3.61± 0.35 Aab	7.16 ± 0.81 Ac	7.48 ± 1.01 Ac	6.13 ± 1.18 Abc	2.86± 0.42 Aa	0.038	0.035	0.041

different letters a, b, c horizontally and A, B vertically indicate significant differences (*P* ≤ 0.05) between the groups.

The results in Table 6 show no significant effect (*P* > 0.05) of adding flaxseeds powder to sheep diets on the weights of newborn lambs. The average weights in the experimental groups T1, T2, T3, and T4 on the first day of birth were *3.74*, 3.93, 4.24, and 4.30 kg respectively, and after 7 days the average weights were 6.82, 6.91, 7.05, and 7.35 kg respectively, while the average weights of lambs after 15 days were 8.93, 9.19, 9.32, and 9.54 kg respectively.

**Table 6:** Effect of adding different levels of flaxseed powder to feed rations on the birth weights of lambs.

Birth weights (kg)	Experimental groups (Mean ± SD)				*p*-value
	T1 Control Flaxseed (0%)	T2 Flaxseed (3%)	T3 Flaxseed (5%)	T4 Flaxseed (7%)	
At birth	*0.25±3.74*^ns^	*0.12±3.93*ns	*0.17±4.24*^ns^	*0.23±4.30*^ns^	0.89
7 days after birth	6.82*±*0.14 ^ns^	*0.21±6.91*^ns^	*0.13±7.05*^ns^	*0.16±7.35*^ns^	0.72
15 days after birth	*0.22±8.93*^ns^	*0.12±9.19*^ns^	*0.16±9.32*^ns^	*0.23±9.54*^ns^	0.64

ns indicates that there are no statistically significant differences between the means of the groups within one line at the level of (*P* ≤ 0.05).

## 5 Discussion

The results showed a significant effect (*P* ≤ 0.05) of adding flaxseeds powder at a rate of 3, 5 and 7% to the diets of Awassi sheep in increasing the percentage of estrus and the length of the estrus period compared to the control group without addition. This may be due to the high percentages of unsaturated fatty acids in flax seeds, especially Omega-3, which improves estrus and fertility indicators when sheep are fed flax seeds rich in Omega-3 acids. This is due to the stimulation of ovarian activity and the increase in the secretion of reproductive hormones such as oestrogen and progesterone, which control the appearance of estrus signs and their duration (Besharati et al., 2023). This is consistent with the findings of Akhtar et al. (2024) when Marwari ewes were fed diets containing 10%, 15%, and 20% omega-3-rich flaxseed, and also with the results of Didarkhah et al. (2020) when flaxseed powder was added to Baluchi sheep diets at levels of 2%, 5%, 7%, 10%, and 12%.

The results showed a significant increase (P ≤ 0.05) in pregnancy rate, lambing rate, and twin birth rate when flaxseed powder was added at a rate of 3, 5, and 7% to the diets of Awassi sheep. This may be explained by the effect of omega-3 fatty acids in flaxseed powder on the production and secretion of reproductive hormones, which play an important role in improving ovulation rates and fertility (Besharati et al., 2023). This is consistent with the results of Akhtar et al. (2024) when Marwari ewes were fed diets containing different percentages of flaxseed, and is consistent with what Didarkhah et al. (2020) found when flaxseed powder was added to the diets of Baluch sheep, and with the results of Noaman et al. (2022) when 6 and 8% of flaxseed oil were added to the diets of Awassi sheep. This contradicts the findings of Long et al. (2020), when Multiparous Rambouillet ewes were fed diets containing 21% ground flaxseed.

The study results showed that serum oestrogen and progesterone concentrations were significantly affected (*P* ≤ 0.05) by the addition of flaxseed powder to the feed rations. These findings are consistent with those of Akhtar et al. (2024), who fed Marwari ewes diets containing varying proportions of flaxseed powder, rich in polyunsaturated fatty acids. Petit and Twagiramungu (2006) also found that cows supplemented with flaxseed had higher progesterone levels (P4). This increase in progesterone levels may be attributed to an increase in the number of corpora luteum and improved steroidogenesis in the ovaries. Administering fatty acids to sheep during their breeding cycles has been shown to increase blood progesterone levels, as well as increase the size and number of ovarian follicles (Liel et al., 2010). According to Rawlings et al. (1994) and Bartlewski et al. (1999), feeding sheep a diet rich in fatty acids (FAs) increases serum oestrogen concentration (pg/ml) by three to four times as a result of flaxseed containing lignans, which are pre-estrogens that are converted to 17β-estradiol and thus promote the synthesis of sex hormones (Habib et al.*,* 2023; Hutchins & Slavin, 2003). Also, the high content of unsaturated fatty acids in flaxseed also improved the physical characteristics of follicles and ova (Didarkhah et al.*,* 2020; Waleed et al., 2009).

The results indicated no significant effect (*P* > 0.05) of adding flaxseed powder at 3%, 5%, and 7% to sheep diets on lamb weights after birth. This is consistent with the findings of Arif (2016) when flaxseed powder was added to the diets of Karadi lambs at 4% and 8%, and with those of Ji et al. (2026) when Tibetan ewes were fed a concentrated diet containing 8% flaxseed.

## 6 Conclusions

These preliminary findings suggest that dietary supplementation with flaxseed powder may potentially enhance reproductive performance in Awassi ewes by increasing pregnancy rates, twin births, and offspring production. Furthermore, this nutritional intervention appears to elevate serum concentrations of progesterone and oestrogen, while exerting no significant effect on lamb birth weights. However, given the initial nature of these outcomes, further confirmation and large-scale validation are required to definitively establish these effects.

## Ethics declaration

This study was conducted in accordance with the ARRIVE (Animal Research: Reporting of In Vivo Experiments) guidelines. This study was approved by the Faculty of Veterinary Medicine at Hama University. (Approval No (No. 55/2025))

## Author contributions

Researcher dr. Mohamed Alrez wrote the research, conducted the experiments, and prepared and reviewing it for publication, the research. and dr. Rayyan Terro helped in conducting the statistical analysis of the results and tabulating them.

## Declarations

**Ethics approval and consent to participate**: The researchers confirm that the experiment adhered to the European Union Directive on Animal Experimentation (Directive 2010/63/EU) and the ARRIVE guidelines. All animals participating in the experiment were treated according to standard procedures to ensure they were not harmed or disturbed, and all procedures were carried out in accordance with relevant guidelines and regulations. The study received approval from the Scientific and Ethical Committee of the Faculty of Veterinary Medicine at Hama University (No. 55/2025). Informed consent was also obtained from the animal owners for the experiments and the publication of the results, with a commitment to applying best practices in veterinary animal care.

## Consent for publication

Not applicable.

## Availability of data and materials

The datasets used and/or analyzed during the current study are available from the corresponding author on reasonable request.

## Funding

There is no funding for this study.

## CRediT authorship contribution statement

**Mohamed Alrez:** Writing – review & editing, Writing – original draft, Supervision, Resources, Funding acquisition, Formal analysis, Data curation, Conceptualization. **Rayyan Terro:** Visualization, Validation, Investigation.

## Declaration of competing interest

The authors declare that they have no known competing financial interests or personal relationships that could have appeared to influence the work reported in this paper.

The authors declare the following financial interests/personal relationships which may be considered as potential competing interests.
